# eHealth Use Among First-Generation Immigrants From Pakistan in the Oslo Area, Norway, With Focus on Diabetes: Survey Protocol

**DOI:** 10.2196/resprot.5468

**Published:** 2016-04-25

**Authors:** Naoe Tatara, Marte Karoline Råberg Kjøllesdal, Jelena Mirkovic, Hege Kristin Andreassen

**Affiliations:** ^1^ Department of Computer Science Faculty of Technology, Art and Design Oslo and Akershus University College of Applied Sciences Oslo Norway; ^2^ Department of Community Medicine, Institute of Health and Society Faculty of Medicine University of Oslo Oslo Norway; ^3^ Center for Shared Desision Making and Collaborative Care Research Oslo University Hospital HF Division of Medicine Oslo Norway; ^4^ Norwegian Centre for eHealth Research University Hospital of North Norway Tromsø Norway

**Keywords:** immigrants, diabetes, information-seeking behavior, inequality

## Abstract

**Background:**

A variety of eHealth services are available and commonly used by the general public. eHealth has the potential to engage and empower people with managing their health. The prerequisite is, however, that eHealth services are adapted to the sociocultural heterogeneity of the user base and are available in a language and with contents that fit the users’ preference, skills, and abilities. Pakistani immigrants in the Oslo area, Norway, have a much higher risk of Type-2 diabetes (T2D) than their Norwegian counterparts do. In spite of having access to information and communication technology (ICT) and the Internet, ICT skills in this population are reported to be relatively low. Further, there is insufficient information about their use of and attitudes toward eHealth services, necessitating investigation of this group in particular.

**Objective:**

This study targets first-generation immigrants from Pakistan living in the Oslo area and examines their use of and attitudes toward eHealth services, specifically: information searches, communication using ICT, and use of ICT for self-management or decision making, all concerning T2D.

**Methods:**

Due to a high prevalence of low literacy among the target population, we employed questionnaire-based individual interviews. The questionnaire was developed by implementing potentially relevant theoretical constructs (technology acceptance model (TAM) and health belief model (HBM)) as measures. To explore issues around language, culture, and general ICT skills, we also implemented questions that we assume were particularly relevant in the context studied but do not appear in any theoretical frameworks. The questionnaire was revised to reflect results of a pilot study involving 10 participants. We employed culturally sensitive sampling methods to reach informants who could otherwise fail to be included in the survey.

**Results:**

This paper presents a survey protocol. The data collection is ongoing. The aim is to collect 200 responses in total by March 2016.

**Conclusions:**

For eHealth to become an influential social innovation, equal access to eHealth services regardless of users’ language, culture, and ICT skills is a prerequisite. Results from this study will be of importance for understanding how people who may not maximally benefit from eHealth services today could be targeted in the future.

## Introduction

### Use of eHealth as a Common Practice

In light of the rapidly increasing number of people having access to Internet and mobile-broadband subscriptions [1], eHealth has a great potential to be a platform for social innovation that mitigates social disparity. eHealth is defined as “all kinds of information and communication technology used for supporting health care and promoting a sense of well-being” [2]. Today, massive amounts of information about health care and well-being are available via the Internet, from multiple sources and in many languages. Studies have shown that using the Internet to seek information relevant to health care and wellness is a widespread practice [3-7]. In Norway, 96% of the population (between 9- and 79-years old) had access to the Internet, 98% to a mobile phone, and 80% to a smartphone in 2014 [8]. In 2015, 62% of the Norwegian population (16- to 79-years old) had sought the Internet for health-related information during the last 3 months [6]. Health-related use of the Internet was highest in the age group 25 to 34, where it reached up to 79%.

Included in eHealth is technology supporting self-care, or “what people do for themselves to establish and maintain health, prevent and deal with illness” [9]. In addition to health-related information searches, the use of information and communication technology (ICT) for the purpose of self-care includes communication with health care providers, peer-to-peer support, self-management by recording and tracking relevant data, and decision making. It is not yet so common to use ICT for these purposes as a Web-based health information search [7,10-12]. However, eHealth for self-care has been gaining increased attention from both the academic research field and consumer health market, especially for chronic diseases, such as diabetes [13-16].

### Immigrants from Pakistan in the Oslo Area – Diabetes and ICT Experiences

One of the advantages of eHealth is that each user can actively choose the eHealth services that best fit their individual needs. eHealth services in the user’s primary language, adapted to cultural preferences, thus appear to be helpful tools. This advantage applies in particular to ethnic minority populations, such as immigrants, to supplement other health services where language and cultural barriers can be of note. In Norway, the number of immigrants has been steadily increasing over the last 40 years. At the beginning of 2015 [17], immigrants and Norwegians born to immigrant parents comprised 15.6% of the whole population. Of those, more than one-half (8.7% of the entire population) originated from Asia, Africa, Latin America, Oceania (except Australia and New Zealand), and Europe (except the European Union) and European Economic Area. Oslo municipality has the largest proportion of immigrants (first-generation immigrants and those born to first-generation immigrant parents) in the country (32%). For example, of first-generation immigrants from Pakistan in Norway, 69.5% live in Oslo [18]. Suburbs of Oslo also have higher proportions of immigrants than the country average [18,19].

A study among four immigrant groups in the Oslo area [20] showed that Pakistanis had the highest prevalence rates of diabetes (women: 26.4%, men: 20.0%). These rates are alarmingly high and much higher than the rates of ethnic Norwegians (women: 2.7%, men: 6.4%). Most of the diabetes cases are Type 2 diabetes (T2D), where leading a healthy lifestyle, including diet and physical activity, plays an important role in self-care. For Pakistani immigrants, barriers to being physically active [21] and maintaining a healthy diet [22] are different from those of ethnic Norwegians. They have also reported experiences of problems in communication with health care providers regarding dietary advice, both due to language barriers and to cultural differences in food traditions [23]. Culturally adapted interventions to the population have been shown to have a positive effect on reducing levels of T2D risk factors [21,24,25].

To date, there is no sufficient information available about the extent to which immigrants with Pakistani background in the Oslo area are using or benefiting from eHealth resources for self-care of T2D, and what their preferences are. Knowledge is scarce regarding their access to and usage of ICT devices and the Internet in general as well. A survey of digital skills and access to ICT among five immigrant groups (Pakistani, Polish, Iraqi, Somali, and Vietnamese) in 2010 [26] showed that 74% of the respondents with a Pakistani background used a personal computer (PC), and 69% also used the Internet. Pakistanis scored lowest among the five immigrant groups surveyed regarding ICT skills. This survey also showed that among Pakistanis, being female, aged over 40, or having an education from only primary school or less were associated with significant reduction in ICT skills scores. Among Pakistani respondents, 56% had an education from primary school or less. Also, the survey report identified that the barriers to strengthening ICT skills were mostly related to knowledge about ICT and lack of available courses, while access to ICT devices or the Internet was little mentioned by the survey participants.

These results suggest that many individuals among immigrants with a Pakistani background may not have been able to benefit from using eHealth services, despite having ICT devices and access to the Internet. Because of a high proportion of the Pakistani population with low educational level [3,19], information written in Urdu may also be inaccessible or difficult to read. We speculate that even those with sufficient ICT skills may have experienced that eHealth services fitting their context are limited compared with those for the ethnic Norwegian population living in the same area. Providing eHealth services that meet the ability and needs of this population, combined with specific training in ICT use, may trigger the motivation for using ICT in general, as well as for health purposes. As Townsend et al [27] argue, “access alone, if not accompanied by services, support, and resources designed to reach and appeal to diverse populations, will not automatically improve an individual’s eHealth use, or their health outcomes” and “issues of equity need to be considered regarding disparity in access to skills, education, and opportunities to develop them.”

The existing knowledge so far [18,26,28] implies that the Pakistani immigrant population is highly heterogeneous when it comes to, among many other factors, ICT skills, literacy levels in their native language, attainment of Norwegian language, attachment to the culture of origin, and acculturation to the environment of Norway. We need to identify subgroups of this demographic group that would benefit most from eHealth use for self-care of T2D and support.

### Aim of the Study and Research Questions

Today there is a digital divide that goes beyond access to ICT resources among Pakistani immigrants in Norway. This gap needs to be addressed to realize the potential in eHealth to mitigate social disparity.

This study aims to provide new insights into the use of and attitudes toward relevant eHealth services to self-care of T2D in this group. Types of eHealth use studied are information searches, communication using ICT, and use of ICT for self-care or decision making. Information searches include using search engines by entering search terms, visiting specific websites to read relevant information, and use of look-up applications. Communication using ICT includes closed communication such as voice or video conversation as well as text messaging, use of social network services, and online consultation to peers or health care professionals. ICT for self-care or decision making includes applications and similar to either record and track data of oneself, such as diet, physical activity, or blood glucose level, or assess user’s health condition or risk based on input data.

The following research questions will be addressed:

1. What are the traits of informants concerning the use of and attitudes toward eHealth for their self-care of T2D?
2. How do informants describe barriers and facilitators of using eHealth services for their self-care of T2D in general?
3. What are their experiences of eHealth services for their self-care of T2D concerning their language and culture?

The survey will help us to break down the problem areas and provide a necessary overview of the status quo in eHealth use for self-care of T2D and attitudes among the target population. Once complete, the study will contribute to defining target users, their requirements and experiences with eHealth services for self-care of T2D.

### Theoretical Framework

It is important to use theoretical frameworks when designing studies on predicting factors of technology use [29]. We, therefore, based our work on well-established theoretical frameworks relevant to ICT use and health behaviors. Many recent studies [30-36] have used technology acceptance model (TAM) [37] and its derivatives [38,39] as a basis to analyze the general public’s or the users’ acceptance of eHealth. TAM has been very well documented and widely used to analyze users’ acceptance of ICT-based tools. TAM predicts users’ acceptance of technology by behavioral intention (BI) to use the technology. BI is explained by users’ perceived ease of use (PEOU) and perceived usefulness (PU). PU is partly explained by PEOU. The studies using TAM to analyze acceptance of eHealth typically attempt to extend TAM to find out antecedent factors of BI, PEOU, and PU or combine with other models.

Tested antecedent factors on the user side are mainly categorized into personal factors, organizational factors, and social factors. Personal factors include factors related to being a patient (or ones relevant to the purpose of using eHealth), to being an ICT user, and factors potentially related to both issues such as sociodemographic variables. Organizational factors include users’ relationship with health care personnel or institute, users’ satisfaction with them and available support in using technology. Social factors mostly refer to extrinsic motivation by others.

Given that use of eHealth has the potential to enhance or trigger positive health behaviors, if not improve health condition or prevent diseases by itself, it is reasonable to assess eHealth use as a component of relevant health behavior theories (HBTs). A well-known model of individual health behavior is the Health Belief Model (HBM) [40]. In HBM, individual behaviors are predicted by individual beliefs and triggered by cues to those behaviors. For example, it is reasonable to see searching the Internet for health information as a health behavior in HBM because it increases health knowledge. An integrated model of TAM and HBM has been tested and proven to be able to predict Internet use for health purpose by working women living in an urban area of Malaysia [35].

In this study, we do not intend to suggest a new model of eHealth acceptance or to test the applicability of existing models to our settings. Instead, we use relevant established theories as a framework to investigate eHealth use and to identify users’ background traits depending on eHealth use and attitudes to it. Therefore, we implemented questions that supplement measures in the theoretical frameworks to explore areas that those frameworks do not cover. Such questions are especially concentrated on cultural and language oriented traits of the target population.

## Methods

### Sample and Recruitment

We are particularly interested in how language barriers and cultural background of the target population are associated with their eHealth use for self-care of T2D. Due to this focus, we employ purposive sampling and use culturally adopted recruiting methods to assure inclusion of otherwise “hard-to-reach” informants. The following inclusion criteria were set: (1) immigrated from Pakistan after the age of 18 and living in the Oslo area, (2) speak Urdu (the official language of Pakistan) as the primary language in their private life, (3) are aged between 25 and 59, (4) have access to or interest in ICT-tools (PC, tablet, or smartphone), connected to the Internet in daily life, and (5) are motivated for and capable of activities for self-management or prevention of T2D.

Before finalizing the inclusion criteria and the questionnaire to use, we carried out a pilot testing with 10 informants who satisfied all the inclusion criteria above but “after the age of 18” in the first criterion. The pilot testing included two informants who immigrated to Norway at an early age. Although they claimed that their primary language was Urdu, they had received primary educations in Norway and did not have any difficulties in using ICT and eHealth in the Norwegian language. Given the fact that many Pakistani people immigrate by marriage to Pakistani-Norwegians [18], we decided to add age at the time of immigration to 18 and above. The lower age limit in the third criterion aligns inclusion criteria employed in previous intervention studies for prevention of T2D among the Norwegian-Pakistani population. [21,22,24,25].

The upper age limit and the last two inclusion criteria are set to highlight issues of language barriers and cultural differences beyond the access to ICT by eliminating common issues between the target population and the ethnic Norwegian population. Figures from 2010 show a significant drop in the percentage of the Norwegian population over the age of 55 that use the Internet to seek health-related information [6]. The proportion of the population using the Internet for such purpose among those aged between 55 and 64 increased by 20 percentage points in 2013, so upper age limit of 59 in 2015 and 2016 is reasonable.

Regarding the last criterion, activities in concern include having a healthy diet and being physically active. We employ a question asking about informant’s intention to change lifestyle for self-care of T2D used in a relevant study [25]. The question is related to trans theoretical model [41]. We include those who consider changing behavior or already have done so as informants. Although we use this question as an inclusion criterion, an answer to it corresponds to a stage of lifestyle change and is an important personal factor related to his/her health. Therefore, we will include a given answer to this question in the analysis of results.

For recruitment, we follow multirecruitment strategies recommended from the experience of difficulties in recruiting South-Asian people [42]. Intervention studies among the Norwegian-Pakistani population also used these strategies [21,22,24,25]. Two research assistants who have an established connection to the Norwegian-Pakistani community in the Oslo area will be in charge of recruitment. Due to expected low literacy level, recruitment is done verbally, by phone calls and house visits in areas with high proportions of Pakistani immigrants (mainly southeast of the Oslo area [18]). A written invitation is used additionally. The recruitment strategy includes snowball sampling [43] where people who are invited to the study also contribute to disseminate the study through their networks.

We aim to recruit 200 informants in total for the survey. We consider this sample size is reasonable given the project budget and the following reasons. Sample sizes varied a lot in relevant studies that quantitatively assessed acceptance of an eHealth service using theoretical frameworks: 101 [34], 132 [32], 163 [30], 250 [31], 293 [35], and 1071 [33]. Besides two studies [30,34] where informants were selected among all the individuals having access to the eHealth service of their interest, justification of sample size was not shown. Sampling methods also varied among these studies. A study of users’ perception of social media as a part of chronic disease management also found the situation similar and set the aim of its sample size as 200 to 250 [44]. The informants will receive compensation of a gift card with a value of 500 Norwegian Krone (approximately US$60) at the completion of the survey.

### Data Collection – Survey by Questionnaire-Based Interview

We collect data in the form of questionnaire-based structured individual interviews. One research assistant asks questions orally, and the other assistant fills out the orally given answers on an answer sheet. Answers to open questions are written down in English by one assistant, and the other assistant assures the expression in English is reasonable.

The primary reason for using individual interview was that low-literacy and -education level is prevalent among the target population. This method has the potential to increase response rate as well as provides possibilities to explain questions and alternative answers for closed questions to assure the quality of data. To keep the condition of data collection consistent, we decided to use only the form of individual interview for all the informants regardless of their literacy levels.

### Survey Questions

**Figure 1:**
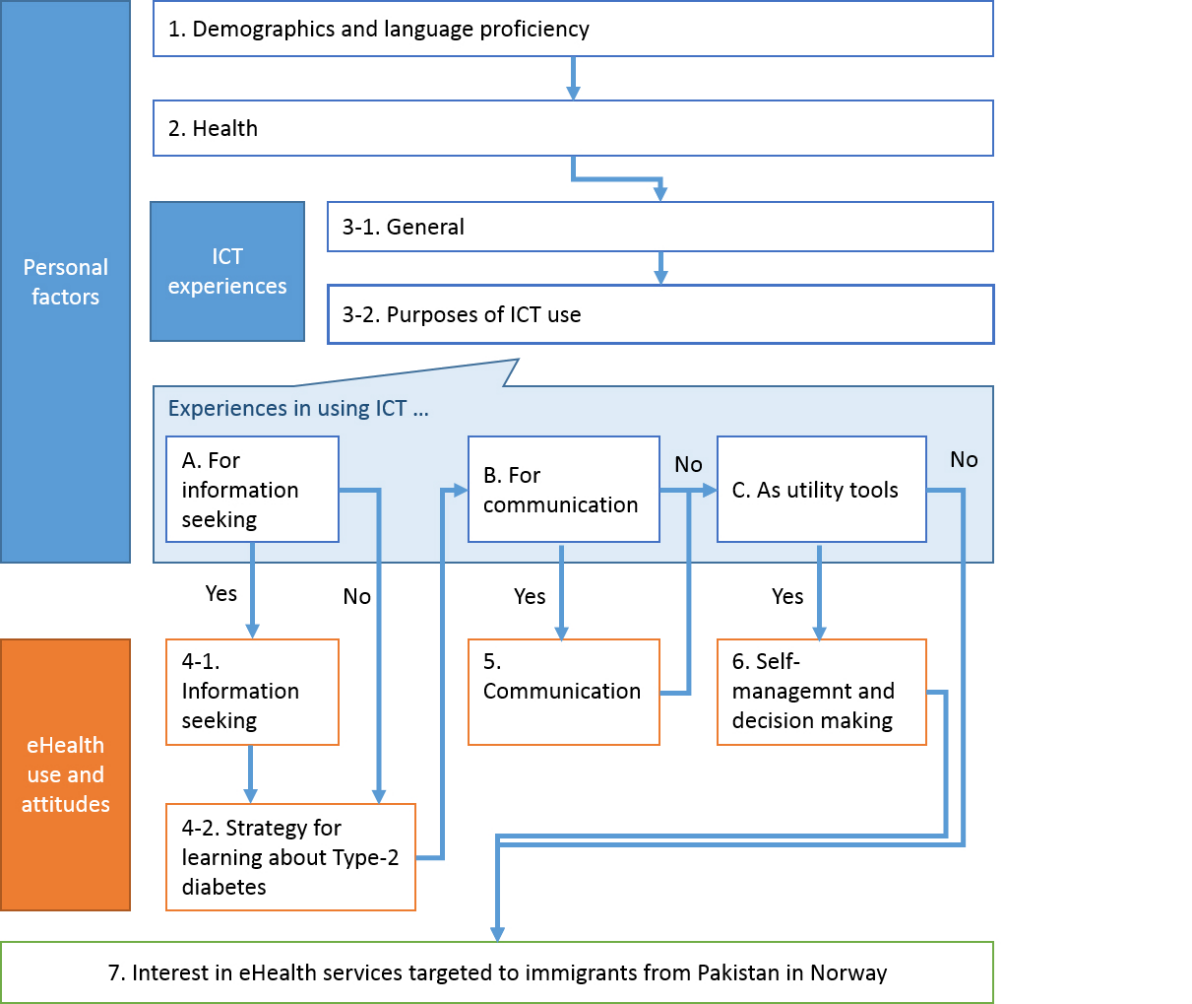
Structure of the questions to use in the survey.

Figure 1 shows the structure of the designed survey questionnaire. They are divided into three broad categories: personal factors (sections 1-3 in Figure 1), experiences of using eHealth services and attitudes toward them (sections 4-6 in Figure 1), and interests in eHealth services targeted to immigrants from Pakistan in Norway (section 7 in Figure 1).

Questions were first formulated in English by the research members. Our approach to developing the questions in the Urdu language was concept driven [45]. We had a workshop where research members and the research assistants who are fluent in both English and Urdu confirmed concepts of questions. After the workshop, the two assistants independently developed questions in Urdu. They confirmed consistency of expression of questions in Urdu language or discussed until they reach consistent results.

In the pilot testing, we tested the questions expressed in the Urdu language and the agreed procedure of the survey. We iteratively improved the questions and the question structure. By the sixth informant in the pilot testing, the questions and the question structure were finalized.

Multimedia Appendix 1 shows the entire set of the survey questions. The final version of the questionnaire consists of seven sections and 102 main questions in total. The number of questions an informant will answer is fewer due to the logic tree structure. All the questions asked by Likert-scale employed a 5-point scale. This decision reflects the feedback from the pilot study and Dawes’ study [46] showing that there is very little difference in data characteristics between 7- and 5-point Likert scales.

#### Personal Factors

Questions under this category are divided into three sections corresponding to three subcategories: demographics and language proficiency (section 1), health (section 2), and ICT experiences (section 3).

The “demographics and language proficiency” subcategory includes an educational level in both Pakistan and Norway, the period of being in Norway and their confidence in writing and reading in Urdu script, Roman Urdu, and Norwegian. Roman Urdu is the Urdu language written in Roman letters. We included Roman Urdu because the pilot study revealed that Roman Urdu is often used among the target population, especially for text communication using ICT devices.

The “health” subcategory includes measurement items for theoretical constructs within relevant HBTs. We considered various validated measures used in relevant studies [24,30,33-35,38,47-49] and agreed on employing measures of theoretical constructs that were shown to predict BI of the studied eHealth service directly or indirectly. Included measures are; self-efficacy (adopted from [49]), perceived health risk, and health consciousness (adopted from [35]), health anxiety and optimism (adopted from [33]), and health knowledge scale (adopted from [30]). For knowledge about risk factors, we based our questions on an intervention study to the Pakistani-immigrant women at a high risk of T2D in the Oslo area [24]. Besides them, we have employed measures concerning their relationship with health care providers from [50] as organizational factor explained in the “theoretical framework” section above.

The “ICT experiences” subcategory is further divided into two parts. The first part, subsection 3-1, consists of questions asking about access to ICT in general including indirect access. Here, indirect access means that another person operates ICT devices on behalf of an informant. The second part, subsection 3-2, consists of questions asking informant’s experience in using ICT for different types of purpose that correspond to the types of eHealth services in this study. Therefore, these questions serve as a filter to ask further questions about the use of eHealth service.

#### eHealth Use and Attitudes

This category is divided into three sections corresponding to the three types of eHealth services the study is addressing (information searches (section 4), communication using ICT (section 5), and use of ICT for self-management or decision making (section 6)). All the questions asking about experiences of using ICT include subquestions asking which languages informants use for each purpose. The informants in the pilot testing used a variety of methods for both text typing/input and reading. Therefore, we implemented subquestions asking about methods to use for each purpose of ICT use of our interest. To those who have experiences of using the specific types of eHealth services, we prepared an open question to ask about the experiences in detail. For each open question, we prepared a series of keywords and phrases as hints to make it easier for informants to remember the experiences. The key words and phrases include among others content fitness to informants’ culture, lifestyle, language, gender, and age, concern about information security and privacy, and trustworthiness of services. We employed this way to explore any relevant issues to their eHealth use originating from their personal background rather than increasing the number of questions to ask informants about potentially irrelevant issues. During the pilot study, we identified several cases that theoretical constructs within TAM and its derivatives were not applicable to explain not using eHealth services. Thus, we also included questions asking reasons for not using each type of eHealth services as well as their attitudes toward using the eHealth services.

For section 4, the subcategory of information searches, we added a group of questions (subsection 4-2) that ask about informants’ strategy for learning about T2D regardless of using ICT. These questions will be asked regardless of the experience of ICT use for any information searches in general.

#### Interest in eHealth Services Targeted to Immigrants from Pakistan in Norway

Section 7 includes measurement items for theoretical constructs within TAM and its derivatives. These measure are BI, PEOU (adopted from [33]), PU (adopted from PU2, PU3, and PU4 in [38]), subjective norms (adopted from SN1 in [38]) and intrinsic motivation (adopted from [33]), and image (adopted from IMG2 in [38]). The technologies in concern for these measures are the three types of currently available eHealth services for self-care of T2D. Due to the difficulty in differentiating expressions in Urdu language and the reported frustration of being asked very similar questions more than once in the pilot testing, we decided to ask one question for a measure of BI, subjective norms, and image. While we employed open questions to ask the reasons for no experience of using eHealth services in the previous sections, we implemented an open question to ask what would make them interested in using each type of eHealth service in a constructive manner. Also, we designed two questions that ask level of their interest in eHealth services designed for the target population in this study and in being involved in the design process of such services. Finally, we set questions asking their awareness of the Norwegian Diabetes association’s services provided in the Urdu language including eHealth services and the level of their interest.

### Analysis

The data analysis process will start by examining the validity of measurement models and measurements by using confirmatory factor analysis. Due to a potential for high heterogeneity of given answers and the inclusion of originally formulated questions, we will primarily employ a qualitative approach in data analysis and corroborate with results of quantitative data analysis. We will use descriptive statistics first to find out central tendencies. We will carefully examine the distribution of data for applicability of parametric analysis. If a parametric analysis is not suitable, we will use a nonparametric analysis. Because it is very likely that informants’ backgrounds vary a lot, we expect that answers given to open questions will provide hints of unforeseen factors that may partly explain theoretical constructs. Thus, we will follow the framework of thematic analysis proposed by Braun and Clarke [51] to identify repeatedly emerging themes at the semantic level.

### Ethical Approval

This study collects sensitive data such as diagnosis of T2D and educational levels in Pakistan and Norway. Even though the study does not collect any directly identifiable personal data, a relatively small sample size in a limited area of Norway and the inclusion criteria may compromise their anonymity. Ranges rather than exact values were used to record information regarding the informants’ age and the period of being in Norway.

At the recruitment, the research assistants explain informants the purpose of this study, what types of questions are included in the survey, that the data is handled anonymously and kept in secured storage, who will attend the interview, and who will have access to the anonymized data. Informants will receive this information in a written format as well at the beginning of an interview. Informed consent is given verbally, which is allowed by the institutional review board, the Norwegian Social Science Data Services (NSD) [52]. The project protocol including the ethics of sampling and recruitment procedures was approved by NSD in June 2015 (project number: 43549).

## Results

This paper is a protocol paper and data collection from the main survey is still in progress at the time of submission. The main survey started in September 2015. By the end of November, we collected responses from 103 informants. The expected time to complete data collection is the end of February 2016.

## Discussion

### Challenges to Include Immigrant Populations

This paper presents the protocol of a survey on eHealth use concerning T2D among first-generation immigrants from Pakistan in the Oslo area. Our focus is on their eHealth experience in relevance with language barriers and cultural differences. Although eHealth has a great potential for immigrant populations for easy access to culturally sensitive health information, knowledge on status-quo of eHealth use in immigrant populations is scarce [7,12,53-56]. Also, such knowledge cannot be generalized because of the diversity of immigrants, including the combination of their country of origin and the country they immigrated to. Failure in involving immigrant populations in research studies is not limited to surveys, which often requires literacy in the language of the survey, but is also common in clinical intervention studies in general unless they specifically target such populations [42,57]. Ensuring inclusion of immigrant populations requires culturally sensitive methods regarding recruitment and data collection. We have adopted such a strategy, aiming especially to include those who may be excluded or may feel reluctant to participate if we took other methods, such as a Web- or paper-based survey with probability sampling and invitation by letter only [58,59]. However, our chosen methods have the challenges and limitations.

First of all, the methods for recruiting and data collection are time-consuming and resource-demanding. Due to the language, the methods rely on the Urdu-speaking research assistants who recruit informants and collect data. One interview takes 1 to 1.5 hours, so the number of interviews per day is limited.

Secondly, informants’ background variables, such as gender and age range, potentially have a skewed distribution. This challenge is not only related to chosen methods, but to general challenges in the group and other. From very early phases of the study design, we were aware of a potential challenge for recruiting male participants. Our research assistants have relevant experiences from an intervention study to prevent T2D among women with Pakistani background in the Oslo area (InnvaDiab study) [25,60]. Thus, their connection to the female society is strong but they have a weak connection to male society. Further, we were informed of a general tendency that Pakistani men do not feel comfortable being asked personal questions and talking about their health. A large proportion of Pakistani men are engaged in shift-time work in transportation service [18], work extraordinarily many hours, and have little flexibility. We will try to overcome such challenges by offering interviews at various times of the day as well as on weekends. A higher response rate by female than male informants is observed in other studies with similar context as well [12,30,44,61]. This gender unbalance may be an interesting finding by itself for further discussion, especially in the light of reported lower education, lower employment rates, poorer integration, and more health concerns among women than men among Pakistani immigrants [28]. Higher participation by women than men in our survey may be a consequence of inclusion criteria, especially regarding interest in prevention and self-care of T2D. Therefore, while we will do our best to recruit informants with balanced gender and age distribution, we prioritize reaching to the aimed sample size. Our study will shed light on challenges with including both genders from the Pakistani immigrant population, which can be useful for future studies.

Lastly, we need to note a potential risk of selection bias, where informants’ personal factors may differ from the represented population. Because we do not have our sample ready yet, we refer to a reliability of the sample in the InnvaDiab study [25,60] recruited in the same way to justify our methods. In the InnvaDiab study, the sample was comparable to samples in other studies among Pakistanis in Oslo using other methods [62], and to data from Statistics Norway [28,63], concerning weight, education level, the number of children, and length of stay in Norway. With the mentioned challenges and limitations in mind, we aim to reach a representable sample of the target population and provide an adequate overview of their eHealth use.

### Conclusions

For eHealth to be truly a social innovation, it should be readily accessible and useful regardless of users’ ethnicity, country of residence, or primary language. This is in line with a claim by World Health Organization [64]. Van Gemert-Pijnen and colleagues [2] propose a holistic framework for a development of eHealth technologies, the Center for eHealth Research and Disease Management roadmap. The roadmap highlights a contextual inquiry as the first activity in a process of gathering information about intended users and the environment where the technology will be implemented. Although such a contextual inquiry presumes who the “intended users” are, a demographic group of “Pakistani immigrants in the Oslo area” is neither sufficient nor appropriate as a definition of the “intended users” to address the issue of digital divide. Results of this study will identify the intended users by highlighting the importance of understanding how people who may not benefit from eHealth could be targeted in the future. The results will then further inform the design of a contextual inquiry study that will be used to design, develop, and disseminate eHealth services for self-care of T2D adapted for the intended users.

The study will also be of importance to research and policy makers that aim to mitigate the social disparity in health among immigrants. In these regards, the study design described in detail in this article will be valuable in providing a basis for designing a survey of eHealth use by immigrants in other contexts.

## Appendix

Multimedia Appendix 1 Survey questionnaire.

## Abbreviations

BI: behavioral intention
HBM: health belief model
HBTs: health behavior theories
ICT: information and communication technology
NSD: Norwegian Social Science Data Services
TAM: technology acceptance model
T2D: type-2 diabetes
PC: personal computer
PEOU: perceived ease of use
PU: perceived usefulness
SMS: short message service
