# Supplementary material for: eHealth Use Among First-Generation Immigrants From Pakistan in the Oslo Area, Norway, With Focus on Diabetes: Survey Protocol
Source: JMIR Res Protoc. 2016 Apr 25;5(2):e79. doi: 10.2196/resprot.5468 (PMC4873306; doi:10.2196/resprot.5468)
Supplement: Multimedia Appendix 1 [file resprot_v5i2e79_app1.pdf]

0. Inclusion Criteria

Only those whose answers are yellow-marked ones are included in the study.

1. Did you immigrate from Pakistan after the age of 18?

   ☐ Yes ☐ No

2. Is Urdu your primary language in your private life? (Do you feel most comfortable and confident in using Urdu among the languages you can use?)

   ☐ Yes ☐ No

3. Have you thought about changing your lifestyle to prevent or for self-management of diabetes? [1]

   ☐ I have not changed my lifestyle the last 6 months, and I am not planning to for the next 6 months.
   ☐ I have not changed my lifestyle the last 6 months, but I am considering doing so within the next 6 months.
   ☐ At the moment, I am trying to change my lifestyle, but not on a regular basis.
   ☐ During the last 6 months, I have changed my lifestyle.
   ☐ I have changed my lifestyle more than 6 months ago.

4. Are you using or interested in using digital tools such as computer, smartphone or tablet computer that are connected to Internet in your daily life (for example; at home, at work, at school or education center you go daily)?

   ☐ Yes ☐ No

5. (For stratification) Which year were you born in?

   ☐ 1956 – 1970 ☐ 1971 – 1980 ☐ 1981 - 1990

6. (For stratification) What is your gender?

   ☐ Male ☐ Female

1. Personal Factors – Demographics and Language Proficiency

1. What is the highest grade or level of schooling you completed in Pakistan?

   ☐ Not completed primary education (5 years from age of 5)
   ☐ Primary education (5 years)
   ☐ Junior secondary education (3 years)
   ☐ Secondary school / technical training college / vocational institutes (2 years)
   ☐ Higher secondary (intermediate) school / polytechnics / commercial institutes (2-3 years)
   ☐ College graduate (Undergraduate / bachelor) (2-5 years)
   ☐ Postgraduate

2. Regarding your Urdu-language proficiency, please answer to which extent you can agree on the following statement. Answer by 5-point Likert scale. 1: Strongly disagree, 2: Disagree, 3: Neutral, 4: Agree, and 5: Strongly agree.

   1a. Reading Urdu in Urdu alphabet.
   1b. Reading Roman Urdu.
   2a. Writing Urdu with Urdu alphabet.
   2b. Writing Roman Urdu

3. How many years have you been in Norway?

   ☐ Less than 1 year
   ☐ Less than 5 years ($1 \leq x < 5$ years)
   ☐ Less than 10 years ($5 \leq x < 10$ years)
   ☐ More than 10 years

4. How long have you taken Norwegian language course? (e.g., if a participant is now taking the 2nd semester or quitted in the middle of 2nd semester, check "1.0 year (2semester) or less")

   ☐ Never
   ☐ 0.5 year (one semester) or less
   ☐ 1.0 year (2 semesters) or less
   ☐ 1.5 year (3 semesters) or less
   ☐ 2.0 years (4 semesters) or less
   ☐ More than 2.0 years

5. Have you ever been in Norwegian schooling system (NOT language school)?

   ☐ Yes ☐ No -> Jump to Q7

6. What is the highest grade or level of schooling you completed in Norway?

   ☐ Primary school
   ☐ High school
   ☐ Post high school training other than college (vocational or technical)
   ☐ Some college or higher
   ☐ I go to school/college now but have not completed.
      ➢ Specify the level: ___________________________

7. Regarding your Norwegian-language proficiency, please answer to which extent you can agree on the following statement. Answer by 5-point Likert scale. 1: Strongly disagree, 2: Disagree, 3: Neutral, 4: Agree, and 5: Strongly agree.

		1	2	3	4	5
1	I am very confident in speaking Norwegian (specify Bokmål or Nynorsk).					
2	I am very confident in reading Norwegian.					
3	I am very confident in writing Norwegian.					

8. Is there any other languages you are confident in reading? If any, please specify.

   ☐ Yes

   ➢ Language(s): ____

   ☐ No

2. Personal Factors – Health

1. Have you ever been diagnosed as having Type-2 diabetes?

   ☐ Yes ☐ No

2. In general, would you say your health is… [2]

   ☐ Excellent
   ☐ Very good
   ☐ Good
   ☐ Fair
   ☐ Poor
   ☐ Going up and down

3. To which extent do you agree that the following statements describe you or your belief? Answer by 5-point Likert scale. 1: Strongly disagree, 2: Disagree, 3: Neutral, 4: Agree, and 5: Strongly agree.

Self-efficacy [3]

		1	2	3	4	5
1	I feel confident in my ability to manage my life-style in terms of prevention/self-care of diabetes.					
2	I am now capable of doing necessary activities in terms of prevention/self-care of diabetes. (NB! Here "capable of" means that you are in a condition where you can do whatever you want to, e.g., not in a health condition that prohibits physical activity; not in a condition where s/he cannot choose what to eat, how much to eat or when to eat, etc.)					
3	I am able to do my own routine activities in terms of prevention/self-care of diabetes.					
4	I feel able to meet the challenge of controlling my life-style in terms of prevention/self-care of diabetes.					

Perceived susceptibility to diabetes [4] (except 6b)

		1	2	3	4	5
5	I would say that I am the type of person who is likely to get diabetes.					
6a	There is a person with diabetes among my family members. (!NB: Here “Family members” include parents, grand parents and siblings.)					
6b	Given the family history of diabetes, I [have / had] a strong chance to get diabetes					
7	I [have / had] a strong chance of getting diabetes due to improper daily habits (drinking, smoking, dietary habit, lack of exercise, etc).					
8	I [am / was] afraid of getting diabetes					
9	[If I get / Due to my] diabetes, I [will have / have] difficulty with my work life (or domestic affairs).					
10	[If I get diabetes, it will hinder / My diabetes hinders] my personal relationships.					
11	[If I get / Due to my] diabetes, I will be long haunted by resultant problems.					

Health anxiety [5,6]

		1	2	3	4	5
12	I'm worried about how healthy my body is.					
13	Thinking about my physical health leaves me with an uneasy feeling.					

Health optimism [5,6]

		1	2	3	4	5
14	I expect that my health will be excellent in the future.					
15	I believe that the future status of my physical health will be positive.					

Beliefs about diabetes (adopted from [2])

		1	2	3	4	5
16	It seems like everything causes diabetes.					
17	There [is / was] not much I can do to lower my chances of getting diabetes.					
18	There are so many different recommendations about [preventing /self-management of] diabetes, it's hard to know which ones to follow.					

Health consciousness [4]

		1	2	3	4	5
19	I think that I take health into account a lot in my life.					
20	I think it is important to know well how to stay healthy.					

Health knowledge scale [7]

		1	2	3	4	5
21	I am very knowledgeable regarding prevention/self-care of diabetes.					
22	I understand my health problems and how to care for them.					

4. Do you know something that can increase your risk of getting diabetes? (Tick off the items that the participant mentioned.) [8]

☐ Little physical activity
☐ Overweight
☐ Too much sugar
☐ Too much fat
☐ Unhealthy food/drinks
☐ Stress
☐ Family history
☐ Others (Specify:)
☐ Don’t know

5. Do you know any foods that are beneficial to include in the diet in order to prevent diabetes (Tick off the items that the participant mentioned) [8]

☐ Legumes
☐ Fish
☐ Fiber
☐ Vegetables
☐ Others (Specify:)
☐ Don’t know

6. The following questions are about your communication with all doctors, nurses, or other health professionals you saw during the past 12 months.

   1. Have you had any communication with doctors, nurses or other health professionals during the past 12 months?

      ☐ Yes ☐ No -> Q7

   2. How often did they do each of the following? Answer from the following options. 1: Never, 2: Seldom, 3: Sometimes, 4: Usually, and 5: Always. [2]

		1	2	3	4	5
1	Give you the chance to ask all the health-related questions you had.					
2	Give the attention you needed to your feelings and emotions.					
3	Involve you in decisions about your health as much as you wanted.					
4	Make sure you understood the things you needed to do to take care of your health.					
5	Explain things in a way you could understand.					
6	Spend enough time with you.					
7	Help you deal with feelings of uncertainty about your health or health care.					

7. Which language do **they** use to communicate with you (no matter if you use an interpreter or not)?

   1. Doctor(s) ____

   2. [If applicable] Nurse(s) ____

   3. [If applicable] Other health professional(s) ___________________________

8. Do you use interpreter to communicate with doctor(s), nurse(s), and/or other health care professional(s)?

   ☐ Always or most of the times (-> Q9)
   ☐ Sometimes or for limited occasions (-> Q9)
   ☐ Never or not anymore (-> Q10)

9. (If either "Always of most of the times" or "Sometimes or for limited occasions" at Q8) How often do you use the following persons as your interpreter? Answer from the following options. 1: Never, 2: Sometimes or for limited occasions, 3: Always or most of the time.

		1	2	3
1	Family member			
2	Interpreter arranged as public service			
3	Other (Specify).			

10. Have you ever participated in support group for prevention or self-management of diabetes in general? (e.g., Motivation group by Diabetes association, other self-organized group, any research project)

    ☐ Yes ☐ No

11. Have you ever talked about your experiences relevant to prevention or self-management of diabetes (including diet or physical activity) or your status related to diabetes (including your feeling or experiences of symptoms due to diabetes) with your family or friends **without using ICT-tools**?

    ☐ Yes ☐ No

12. Have you ever kept track of information about anything relevant to diabetes (including blood glucose level, diet, physical activity, medication) **without using ICT-tools**?

☐ Yes ☐ No

3. Personal Factors – ICT experiences

3-1: General ICT experiences

1. Do you own any of the following? Mark all that apply and answer which language is used for its Operation System (OS)

If all of these are marked, go to Q3

- ☐ Tablet computer like an iPad, Android Tablet, Windows Tablet and similar connected to Internet
  - ➢Language:
- ☐ Smartphone, such as an iPhone, Android, Windows phone connected to Internet
  - ➢Language
- ☐ Home computer, such as PC (Windows, Unix, Linux) or Mac, stationary or laptop connected to Internet
  - ➢Language
- ☐ Cell phone **with basic functions such as phone call, address book, and SMS (including cases that the participant deactivates or does not use other functions than these)**
- ☐ I do not have any of the above

2. In the last 12 months, do you use any of the followings **that are not yours** but belonging to family member or are for public use at work, school, or public space (such as a library or NAV) **as a main tool to use Internet**? It includes indirect use (i.e., you ask someone to operate on behalf of you). Mark all that apply.

- ☐ Tablet computer like an iPad, Android Tablet, Windows Tablet and similar
- ☐ Smartphone, such as an iPhone, Android, Windows phone
- ☐ Home computer, such as PC (Windows, Unix, Linux) or Mac, stationary or laptop
- ☐ I do not have access to any of the above
- ☐ I do not use any other devices than mine to use Internet

3. In the last 12 months, how often do you use the followings **by yourself** (whoever the owner is and including use with some help by others)?

		Daily	Less than daily	Less than weekly	Less than monthly	Never used
1	Tablet computer					
2	Smartphone					
3	Home computer					
4	Cell phone without Internet connection					

If «Never used» is marked for all 1-3, Mark "No" at Q7-9 in this section

4. In the last 12 months, how often did you ask somebody else for help to do what you want to do with ICT tools or ask somebody else to operate ICT tools on behalf of you?

- ☐ Always (-> Q5)
- ☐ Often (-> Q5)
- ☐ Sometimes (-> Q5)
- ☐ Seldom (-> Q6)
- ☐ Never (-> Q6)

5. (If the answer to Q4 is either “Always”, “Often” or “Sometimes”) Please specify the reasons why you need help. Mark all that apply.)

- ☐ Lack of reading skills
- ☐ Lack of computer skills in general including typing skill (but having enough reading skills)
- ☐ Difficulty in understanding contents
- ☐ Forbidden direct use of computer/smart phone/mobile phone (due to another owner)
- ☐ Anxiety / Feeling insecure
- ☐ To learn how to do
- ☐ Cumbersome (including “too busy”) to do by yourself (although you have skills and access to do it)
- ☐ Others (Describe)

➔ Go to Q7

6. (IF “Seldom” or “Never” to Q4) Please specify the reasons why you did not ask anybody else to do it on behalf of you.

- ☐ I have enough skills (both computer skills and reading skills) and access to ICT tools
- ☐ I wanted help, but there was nobody who could help around me
- ☐ I wanted help, but it was too cumbersome (including “I was too busy”) to find and/or ask somebody for help
- ☐ People important to me does not like me to use ICT-devices
- ☐ Although I am interested in using ICT-tools, I have never thought about asking somebody else to do what I want to do or asking for help
- ☐ Others (describe)

➔ Go to Q7

3-2: Specific and direct ICT experiences

In the following questions, we would like to ask about **your direct use** of ICT-tools (Tablet computers, Smartphones, or Home computers) divided into three categories;

A. Information search and gaining new information in general (ex., use of search engines, browsing portal sites, use apps for look-up of specific information etc.) regardless of the type of information format (text, media files, etc.)

B. Use of websites / applications for communication (ex. Skype, Viber, WhatsApp, Facebook, Twitter, Instagram, YouTube, Reddit, blogs, etc.), and

C. Use of utility tools, such as applications / software programs that help your daily life or activities (ex. Ruter/NSB app, alarm, timer, calculator, map, camera, note, etc.)

7. First, have you ever used ICT-tools for **information search, look up, and/or gaining new knowledge**? This includes using search engine or portal sites, reading online articles, watching videos, or use of programs / apps to look up information etc.

☐ Yes ☐ No (Skip section 4 Q1-18)

8. Second, have you used ICT-tools for **communication with others**? This includes both synchronous and asynchronous communications, both open and closed communications, and whatever type of communication means to use, but does not include only consuming information made by someone (e.g., only reading someone's blog and not communicating with the blogger).

   ☐ Yes ☐ No (Skip section 5)

9. Third, do you often (more than monthly) use utility tools, such as applications / software programs that help your daily life or activities (other than look-up tools)?

   ☐ Yes -> Which type of the followings?

   - ☐ **Manually recording** any type of information, such as camera, voice record, memo (both hand writing and type text), check list, etc.
   - ☐ Making **inquiry** by setting some condition, such as loan calculator, weather forecast, travel search like "ruter.no", navigation using GPS, etc.
   - ☐ Either totally or partly **automatic function**, such as alarm, reminder, sensor-based information recording etc.
   - ☐ Others (Specify)

   ☐ No (Skip section 6)

10. Other than the three types of purposes to use ICT-tools, what functions / applications / software programs do you use often (more than monthly)?

    For example,

    - Games
    - Media (music (CD, mp3), video (DVD)) player or book reader (ex. Kindle app)
    - Software used at work/study (Microsoft office, Adobe products, GeoGebra, programming etc.)

4. eHealth A - Information for learning about diabetes

4-1. Experiences with use of ICT-tools

In the first part of this section, we are going to ask you about your experiences with use of ICT-tools for getting new information both in general and for learning about diabetes. The questions are divided depending on types of resources you try to get access to information from.

1. (Search, internet, general) Have you used search engine for the Internet such as Google, Yahoo, etc. (including Google voice search and Siri) or any apps to look up information **by using search terms**?

   ☐ Yes ☐ No ->Q7

2. (If "Yes" to Q1) When you do such information search, how often do you take the following ways? Answer from the following options. 1: Never, 2: Seldom, 3: Sometimes, 4: Usually, and 5: Always.

	**Type search terms in**	1	2	3	4	5
A	Urdu alphabet					
B	Roman Urdu					
C	Norwegian and read the results in Norwegian					
D	Norwegian and use Google Translate to read the results in your preferable language ( )					
E	other language ( ) and read the results in the language					
F	other language ( ) and use Google Translate to read the results in your preferable language ( )					
	**Use voice recognition in**	1	2	3	4	5
G	Urdu					
H	Norwegian and read the results in Norwegian					
I	Norwegian and use Google Translate to read the results in your preferable language ( )					
J	other language ( ) and read the results in the language					
K	other language ( ) and use Google Translate to read the results in your preferable language ( )					
L	Other (describe below)					

3. (If "Yes" to Q1) (Search, Internet, **Diabetes**) In the last 12 months, have you used the Internet to **search information relevant to diabetes in general** (including healthy lifestyle for prevention and self-management of diabetes and no matter what types of media format is used) **for yourself** (even with some help by others) **by using search engines with search terms** ?

   ☐ Yes ☐ No (Why?) -> Q7

4. (If "Yes" to Q3) Which methods among the listed ones in Q2 do you take to search information relevant to diabetes in general for yourself?

Frequency	Methods
Always or most of the time	
Sometimes or for limited occasions	

5. (If "Yes" to Q3) Based on your experiences with searching information about diabetes for the Internet by using search terms in the last 12 months, how much do you agree or disagree with each of the following statements? Answer by 5-point Likert scale. 1: Strongly disagree, 2: Disagree, 3: Neutral, 4: Agree, and 5: Strongly agree. [2]

		1	2	3	4	5
1	It took a lot of effort to get the information I needed.					
2	I felt frustrated during my search for the information.					
3	I was concerned about the quality of the information.					
4	The information I found was hard to understand.					

6. (If "Yes" to Q3) What do you think about searching information relevant to diabetes(including healthy lifestyle for prevention and self-management of diabetes and no matter what types of media format is used) for yourself (even with some help by others) by using search engines with search terms? (Hint: difference in quantity or/and quality of information available between used languages, ease of /difficulty in reaching information, finding appropriate search terms, sorting out results, or reading texts, effect on feeling or health behavior, convenience, trustworthiness of information, source of information, etc.)

7. (Only scrolling or clicking, general) Have you used any websites where you basically don't have to type anything but can reach information by just scrolling down, clicking on category, sub-category, and so on, or ticking off some conditions, etc. or subscribe any news e-mail? For example, portal sites (including YouTube), websites of specific organizations or companies, news sites, shopping sites, "finn.no"-type of site, restaurant guide, someone's blogs without intention to write any comments or such etc.,

☐ Yes ☐ No -> Q12

8. (If "Yes" to Q7) When you use such sites or subscription, how often do you take the following ways? Answer from the following options. 1: Never, 2: Seldom, 3: Sometimes, 4: Usually, and 5: Always

	Use sites /applications / subscription written in …	1	2	3	4	5
A	Urdu					
B	Norwegian and read them in Norwegian					
C	Norwegian and use Google Translate to read them in your preferable language					
D	other languages and read them in the language					
E	other languages and use Google Translate to read them in your preferable language					
F	Other (describe)					

9. (If "Yes" to Q7) (Only scrolling or clicking, Diabetes) In the last 12 months, have you used any portal sites, specific websites, blogs, e-mail subscription, etc. to **search or simply gain information relevant to diabetes in general** (including healthy lifestyle for prevention and self-management of diabetes) **for yourself** (even with some help by others)?

☐ Yes ☐ No (Why?) -> Q13

10. (If "Yes" to Q9) Which ways among the listed ones in Q8 do you take to search or simply gain information relevant to diabetes in general for yourself?

Frequency	Methods
Always or most of the time	
Sometimes or for limited occasions	

11. (If "Yes" to Q9) What kind of resources do you use? (Please record the type of language and how the participant read it among the ones chosen in Q10)

☐ Websites of official organizations (such as International Diabetes Federation, WHO, Diabetes association in Norway, Diabetic Institute Pakistan, Directorate of health, etc.) (Language and how to read:)

☐ Related categories in general portal sites or newspaper sites (such as "health and training" in msn.no) (Language and how to read:)

☐ Blogs by an individual (Language and how to read:)

☐ Scientific journals or similar (Language and how to read:)

☐ Others (describe) (Language and how to read:)

12. (If "Yes" to Q9) What do you think about accessing portal sites, specific websites, blogs, e-mail subscription, etc. to get new information relevant to diabetes for yourself? (Hint: difference in quantity or/and quality of information available between used languages, conditional interest depending on information sources, language used, media format, fitness of contents to your lifestyle, culture, gender or age, etc.)

13. (Use of Apps, etc. for lookup, General) Have you used any software programs on PC or apps on Smartphone/tablet by which you can look up information you want? For example, recipes, drug information, dictionaries, etc.

☐ Yes ☐ No

14. (**Search** of Apps, etc. for lookup, **Diabetes**) Have you **searched by yourself** any software programs on PC or apps on Smartphone/tablet by which you can **look up information relevant to diabetes** in general (including healthy lifestyle for prevention and self-management of diabetes) for yourself (regardless of actual installation or use of them)?

☐ Yes ☐ No (Why?) ->Q16

15. (If "Yes" to Q14) Which language did you use to search programs / apps and how satisfied were you with the results in terms of quality of them (i.e., how relevant and seeming useful for your purpose) and efficiency (i.e., how quickly you found programs / apps you were satisfied with)?

		Very satisfied	satisfied	Neither/ neutral	dissatisfied	Very dissatisfied	Never done
1	Urdu						
2	Norwegian						
3	Others						

16. (**Use** of Apps, etc. for lookup, Diabetes) Have you ever **used** any software programs on PC or apps on Smartphone/tablet by which you can **look up information relevant to diabetes** in general (including healthy lifestyle for prevention and self-management of diabetes) for yourself (including the cases where you did not search it but somebody else introduced and/or installed programs / apps for you)?

☐ Yes ☐ No (Why?)
-> If "Yes" to Q14, go to Q18;
If "No" to Q14 , go to Q19

17. (If "Yes" to Q16) What are the software programs / apps and which language is used for them? Please answer how often you use it in the last 12 months as well.

Software program /app	Language (N: Norwegian, U: Urdu If other, specify)	Frequency (5: Daily, 4: Less than daily, 3: Less than weekly, 2: Less than monthly, 1: Not using it anymore)

18. (If "Yes" to **EITHER/BOTH** Q14 **OR/AND** Q16) What do you think about using software programs on PC or apps on Smartphone/tablet by which you can look up information relevant to diabetes in general (including healthy lifestyle for prevention and self-management of diabetes) for yourself? (Hint: difference in quantity or/and quality of programs available between used languages, conditional interest depending on information sources, richness of contents, language used, media format, fitness of contents to your lifestyle, culture, gender, or age, power or memory consumption of the program/apps, how quickly it can be launched, etc.)

4-2. Experiences and strategies for learning about diabetes in general

In the latter part of this section, we are going to ask you about your experience and strategies for learning about diabetes in general regardless of use of ICT-tools.

19. Have you consulted to any resource **without using ICT-tools** (e.g., doctors, diabetes educator, telephone consultation of Diabetes Association, family members, religious leader, friends, patient peers, books, magazine, brochure, etc.) for information about anything relevant to diabetes (including healthy lifestyle, such as recipes for healthy diet and physical activity)?

☐ Yes ☐ No -> Q21

20. (If "Yes" to Q19) Which resources do you consult to for information about anything relevant to diabetes? Please answer frequency to use each resource and language as well.

	Resource	Always or most of the time	Sometimes or for limited occasions	Language
1				
2				
3				
4				
5				

21. Overall, how confident are you that you could get advice or information about diabetes if you needed it (regardless of the information recourses)? Answer by 5-point Likert scale. 1: Very unconfident, 2: Somewhat unconfident, 3: Neither, 4: Somewhat confident, and 5: Very Confident. [2]

13. In general, how much would you trust information about diabetes from each of the following? Answer by 5-point Likert scale. 1: Totally distrust, 2: Distrust rather than trust, 3: Neither / have no idea, 4: Trust rather than distrust, and 5: Totally trust [2]

	Use sites /applications / subscription written in …	1	2	3	4	5
1	Health care professionals (doctor, diabetes educator, nurses)					
2	Family or friends					
3	Official organization such as International Diabetes Federation, Directorate of Health, or Diabetes Association					
4	Religious organizations and leaders					
5	Magazine, news paper, TV					
6	Books, encyclopedia					
7	Internet in general					
8	Scientific journals and similar					
9	Others (if written anything to Q19)					

22. (Open question) Imagine that you had a strong need to get information about diabetes. Where would you go first? [2]

23. Please tell us your strategies regarding learning about diabetes in general (including healthy lifestyle, such as recipes for healthy diet and physical activity) and reasons for them (regardless of means).

    - Any experience with difference in quality or quantity of information depending on language used
    - Any experience regarding fitness of contents to your life style and culture
    - If no experience with ICT-tools to get information about diabetes, what is their attitudes to it and reasons for it, eventually if they are interested in it.

5. eHealth B – Use of ICT-tools for communication with individuals

In this section, we are going to ask you about your experience with using ICT-tools for communication both in general and with focus on topics relevant to diabetes. Questions are divided based on types of communication means and who you communicate with.

1. (Closed, General) Do you often (more than monthly) use ICT-tools for **closed individual communication** (phone call, e-mail, SMS/MMS, Skype, Viber, WhatsApp, Messenger, Google Hangout, etc.)?

   ☐ Yes ☐ No -> Q5

2. (If "Yes" to Q1) Which of the following communication methods do you often (more than monthly) take? Mark all that apply.

   - Synchronous communication
     - ☐ Video communication
     - ☐ Voice communication
   - Asynchronous communication
     - ☐ Text-based communication -> Q3
     - ☐ Use of "emoji", stamps, photos, etc.

3. (If the answer to Q2 includes "Text-based communication") which language and input methods do you use to write text for such individual communication? Answer from the following options. 1: Never, 2: Seldom, 3: Sometimes, 4: Usually, and 5: Always.

		1	2	3	4	5
A	Type Urdu alphabet					
B	Type Roman Urdu					
C	Type Norwegian					
D	Type other language ( )					
E	Use voice recognition in Urdu					
F	Use voice recognition in Norwegian					
G	Use voice recognition in other language ( )					

4. (Closed, Diabetes) In the last 12 months, have you **talked/communicated about your experiences relevant to** prevention or self-management of **diabetes** (including diet or physical activity) or your status related to diabetes (including your feeling or experiences of symptoms due to diabetes) with others **using ICT-tools for closed communication, such as text messages, video or voice calling**?

   ☐ Yes ☐ No (Why?)

5. (SNS, General) Do you often (more than monthly) use applications / web sites for your **social networking** (whatever the purpose of networking is, i.e., including both private and career network. For example, use of Facebook, Pinterest, Reddit, Instagram, Vine, Twitter, Google+, LinkedIn, blogs, tumblr, etc.)?

   ☐ Yes ☐ No -> If "Yes" to Q4, go to Q10; If "No" to Q4, go to Q11

6. (If "Yes" to Q5) How often do you do the followings on applications / websites of social networking service?

		Daily	Less than daily	Less than weekly	Less than monthly	Never done
1	To see updates of others' status and eventually give comments or similar					
2	To update my status and eventually reply to given comments					

7. (SNS, Diabetes) In the last 12 months, have you **shared (as your own post, as a comment to other person's post or as a reply to given comment) your experiences relevant to** prevention or self-management of **diabetes** (including diet or physical activity) or your status related to diabetes (including your feeling or experiences of symptoms due to diabetes) with others **on social networking sites (SNS)**?

☐ Yes -> Q8 ☐ No (Why?)
-> If "Yes" to Q4, go to Q10;
If "No" to Q4, go to Q11

8. (If "Yes" to Q7) Which language do you use to share your experiences relevant to diabetes and how often do you do that?

		Always	Often	Sometimes	Seldom	Never done
1	Urdu					
2	Norwegian					
3	Others					

9. (If "Yes" to Q7) Who could see such posts (shared information)?

☐ Public (i.e., without setting for information sharing)
☐ All those connected on SNS
☐ Limited friends or family members
☐ Others:____

10. (If "Yes" to **EITHER/BOTH** Q4 **OR/AND** Q7) What do you think about sharing your experiences with your family or friends using ICT-based communication services? (Hint: effect of communication on feeling or health behavior, closed/open communication, individual/group conversation, thoughts about privacy and information security issues, who they can think about sharing experiences in terms of relationship, language to use, and cultural commonality, etc.)

11. (Patient-peers, Diabetes) In the last 12 months, have you **used the Internet or mobile applications to communicate with others** (basically NOT family members or friends that the participant has known from before) **that are also in a same health situation with you in terms of diabetes**? (e.g., participating in an online forum/support group such as PatientLikeMe; leaving comments/questions on others' blog/YouTube page)

☐ Yes -> Q12 ☐ No (Why?)
-> Q14

12. (If "Yes" to Q11) Which language do you use for such communication and how often do you do that?

		Always	Often	Sometimes	Seldom	Never done
1	Urdu					
2	Norwegian					
3	Others					

13. (If "Yes" to Q11) What do you think about communicating with such "peers" using ICT-based communication services? (Hint: satisfaction level, effect of communication on feeling and health behavior, trustworthiness of people who s/he has never know from before, value of peers and their real experiences, thoughts about privacy and information security issues (including anonymity), conditional interest (e.g., commonality with peers in terms of culture, language, lifestyle, age, gender,) etc.)

14. (Experts, Diabetes) In the last 12 months, have you **used any service by which you can ask experts (specialists) about prevention/self-management of diabetes** (including healthy diet or physical activity) using Internet, e-mail or mobile applications? (e.g., Diabeteslinjen på nett, Vektklubben, Lommelegen, etc.)

☐ Yes -> Q15 ☐ No (Why?) -> Section 6

15. (If "Yes" to Q14) Which service(s) and language(s) do you use and how satisfied are you with such service?

Service and language (Urdu: U, Norwegian: N, Others: O)	Very satisfied	satisfied	Neither/ neutral	dissatisfied	Very dissatisfied

16. (If "Yes" to Q14) What do you think about using ICT to ask experts about prevention/self-management of diabetes? (Hint: effect of communication on feeling and health behavior, trustworthiness of the experts at online services, how efficiently (quickly) you can get an answer, eventually cost issues, thoughts about privacy and information security issues, conditional interest (e.g., in terms of understanding of own cultural background including lifestyle, foods, used language, age, gender) etc.)

6. eHealth C – Use of ICT-tools as self-help tool

1. (App etc., Advice/Calc./Risk, etc, Diabetes) In the last 12 months, have you used Internet/mobile (App)-based self-help program, i.e., services that **give you automatically generated advices or values based on your data for self-management of your health and well-being**? (e.g., BMI, risk of getting diabetes, recommendation of calorie intake etc., based on your weight, height, blood glucose level, etc.)

   ☐ Yes ☐ No (Why?)

2. (App etc. Tracking etc., Diabetes) In the last 12 months, have you used Internet/mobile (App)-based program to **regularly keep track of your health-relevant information** including diet, physical activity, blood glucose level or medication taking? (e.g., photo of what you ate/drank, step counts, or callorie burnt after physical activity, no matter how such data is made (automatically recorded by smartphone (RunKeeper, Strada, etc.), by a separate sensor such as JawBones, Nike Fuel Band, Polar pulse meter, or by manual input (Diabetesdagboka, GlooKo, etc.)))

   ☐ Yes ☐ No (Why?)
   -> If "Yes" to Q1, go to Q3;
   If "No" to Q4, go to Section 7

3. (If "Yes" to **EITHER/BOTH** Q1 **OR/BOTH** Q2) What are the programs (or if you don't remember the name of the program, the purpose of it) and how satisfied were you with the results in terms of quality of contents (i.e., how relevant and useful for your purpose) and efficiency (i.e., how much effort you made to use it including understanding the returned results)?

Program	Very satisfied	satisfied	Neither/ neutral	dissatisfied	Very dissatisfied

4. (If "Neither/neutral", "dissatisfied", OR "Very dissatisfied" was given to any of Q3) What was the reason you could not be satisfied

5. (If "Yes" to **EITHER/BOTH** Q1 **OR/BOTH** Q2) What do you think about using ICT as a self-help tool for prevention/self-management of diabetes? (Hint: usefulness, effect on feeling and health behavior, accuracy of the tracked data, trustworthiness of automatically generated advices, efficiency compared to manual recording or calculation, eventually cost issues, thoughts about privacy and information security issues on tracked and stored data, conditional interest (e.g., in terms of relevance to own lifestyle, foods, used language, age, gender) etc.)

7. Interest in eHealth services targeted to immigrants from Pakistan in Norway

In this section, we are going to ask about your belief and thoughts regarding three different types of ICT use in the context of prevention and self-management of diabetes (including healthy lifestyle for prevention and self-management of diabetes), namely:

A. Use of ICT-tools for learning (i.e., obtaining information) about diabetes in general. Such activities include search information, reading text, listening audio, or watching videos etc. using ICT-tools, and use of software programs or apps to look up information.

B. Use of ICT-based communication services to communicate with others (including family, friends, peers and experts) in terms of diabetes in general.

C. Use of ICT-based tools for self-help purposes related to diabetes by which you can keep track of your information relevant to diabetes (including what you do for prevention and self-management of diabetes) and/or get automatically generated advices or values based on the recorded information that you can use as a basis of decision-making. This includes use of calculation of for example, calorie intake, energy consumption or BMI, recording of physical activity, diet, blood glucose level or medicine intake, etc.

1. For each of the types of ICT use above, please answer the extent to which you can agree on the following description by; 1: Strongly disagree, 2: Disagree, 3: Neither/don't know, 4: Agree, or 5: Strongly agree.

   If you have no experiences of any type of ICT use, imagine that the descriptions start with "I believe" for the description 2-10.

		A	B	C
1	(BI) I would very actively use ICT for that purpose.			
2	(PEOU) It is very easy for me to use ICT for that purpose. [5]			
3	(PEOU) It is very easy for me to learn how to use ICT for that purpose. [5]			
4	(PU) Using ICT helps me reach information in better quality than other means. [9]			
5	(PU) Using ICT helps me reach information I want more efficiently than other means. [9]			
6	(PU) ICT is useful to achieve that purpose. [9]			
7	(Image) Using ICT for that purpose made me feel that I have a high profile. [9]			
8	(Subjective norms) People who influence the way I behave like that I use ICT for that purpose. [9]			
9	(Intrinsic motivation) Using ICT for that purpose will be/was fun. [5]			
10	(Intrinsic motivation) I like using ICT for that purpose [5]			

2. For each type of ICT use above, what are (or do you think would be) important for you to be interested in using ICT for each purpose?

A – for learning about diabetes	
B – for communicating with others in the context of diabetes	
C – for self-help	

3. Imagine that there is an ICT-based service that serves for purposes of all the three types of ICT-use described above. If that service is specially designed for Pakistani-Norwegian population by involving Pakistani-Norwegian people like you, to which extent do you believe you will be interested in using it?

☐ Very much interested
☐ Interested rather than uninterested
☐ Neither
☐ Uninterested rather than interested
☐ Not interested at all

4. Are you interested in participating in design process of such an ICT-based service so that your voice will be reflected in its design?

☐ Very much interested
☐ Interested rather than uninterested
☐ Neither
☐ Uninterested rather than interested
☐ Not interested at all

5. Did you know that The Norwegian Diabetes association (Diabetesforbundet) offers a variety of services for Pakistani people? For example;

- Diabetes line in Urdu by telephone and on the internet
- Brochures (printed format) written in Urdu
- Webpages written in Urdu
- iOS/Android app (DiabetesID) available in Urdu
- Physical activity instruction video and audio file (mp3) in Urdu
- Visit by an expert
- Information made for immigrants including Pakistanis written/spoken in Norwegian

☐ Yes, I have used them. -> Q6

☐ No, I did not know them. -> Q8

☐ Yes, I know it but have never used them. -> Q8

6. (If "Yes" to Q5) Which services have you used and how satisfied were you?

Service	Very satisfied	satisfied	Neither/ neutral	dissatisfied	Very dissatisfied

7. (If “Yes” to Q5) How did you know the services?

- ☐ From the link "Kan du lite norsk?" on the top page of their website.
- ☐ Searched in their website
- ☐ Used google to find information.
- ☐ Get the URL of information from other persons.
- ☐ It is another person who operated ICT, so I don't know.
- ☐ I don't remember.
- ☐ Others

8. To which extent are you interested in using their services? Please answer by 1: Not interested at all, 2: Uninterested rather than interested, 3: Neither/don’t know, 4: Interested rather than interested, or 5: Very interested.

	Service	Level of interest
1	Diabetes line in Urdu by telephone	
2	Diabetes line in Urdu on the Internet	
3	Brochures (printed format) written in Urdu	
4	Webpages written in Urdu	
5	Physical activity instruction video in Urdu	
6	Visit by an expert	
7	Audio file in Urdu	
8	Information made for immigrants including Pakistanis written/spoken in Norwegian	

9. If you have, please let us hear your opinions about Diabetes association’s services for Pakistani people

Reference

1. Helland-Kigen KM, Råberg Kjøllesdal MK, Hjellset VT, Bjørge B, Holmboe-Ottesen G, Wandel M. Maintenance of changes in food intake and motivation for healthy eating among Norwegian-Pakistani women participating in a culturally adapted intervention. Public Health Nutr 2013 Jan;16(1):113–122. PMID: 22781507

2. National Cancer Institute. Health Information National Trends Survey (HINTS 4), Cycle 3, Next-Birthday, Full-Contents, English version. U.S. Department of Health and Human Services; 2013. http://hints.cancer.gov/docs/Instruments/HINTS_4_Cycle_3_English_Annotated_508c_3_21_2014.pdf. Archived at: http://www.webcitation.org/6dQD2Yr0x

3. Deci EL, Koestner R, Ryan RM. A meta-analytic review of experiments examining the effects of extrinsic rewards on intrinsic motivation. Psychol Bull 1999;125(6):627–668. PMID: 10589297

4. Ahadzadeh AS, Pahlevan Sharif S, Ong FS, Khong KW. Integrating health belief model and technology acceptance model: an investigation of health-related internet use. J Med Internet Res 2015;17(2):e45. PMID: 25700481

5. Svendsen GB, Soholt Y, Munch-Ellingsen A, Gammon D, Schurmann A. The Importance of Being Useful and Fun: Factors Influencing Intention to Use a Mobile System Motivating for Physical Activity. Hawaii Int Conf Syst Sci Los Alamitos, CA, USA: IEEE Computer Society; 2009. p. 1–10. ISBN: 978-0-7695-3450-3

6. Snell W Jr, Johnson G. The Multidimensional Health Questionnaire. Am J Health Behav 1997 Feb;21(1):33–42.

7. Wilson EV, Lankton NK. Modeling Patients' Acceptance of Provider-delivered E-health. J Am Med Inform Assoc 2004 Aug;11(4):241–248. PMID: 15064290

8. Råberg Kjøllesdal MK, Hjellset VT, Bjørge B, Holmboe-Ottesen G, Wandel M. Perceptions of risk factors for diabetes among Norwegian-Pakistani women participating in a culturally adapted intervention. Ethn Health 2011 Jun;16(3):279–297. PMID: 21660786

9. Venkatesh V, Bala H. Technology Acceptance Model 3 and a Research Agenda on Interventions. Decis Sci 2008 May 1;39(2):273–315.
